# Mothers’ willingness to accept and pay for vaccines to their children in western Iran: a contingent valuation study

**DOI:** 10.1186/s12887-020-02208-4

**Published:** 2020-06-23

**Authors:** Satar Rezaei, Abraha Woldemichael, Masoumeh Mirzaei, Shima Mohammadi, Behzad Karami Matin

**Affiliations:** 1 Research Center for Environmental Determinants of Health, Health Institute, Kermanshah University of Medical Sciences, Kermanshah, Iran; 2 Department of Health Systems, School of Public Health, College of Health Sciences, Mek’ele University, Mek’ele, Ethiopia; 3 Student Research Committee, Kermanshah University of Medical Sciences, Kermanshah, Iran

**Keywords:** Willingness to pay, Willingness to accept, Contingent valuation, Childhood vaccination

## Abstract

**Background:**

The clients’ willingness to accept (WTA) and willingness to pay (WTP) for a given good or service can help elicit the monetary value of that good or service. This study aims to assess the WTA and WTP of mothers attending primary health centers for vaccines to their children during 2019 in Kermanshah city, western Iran.

**Methods:**

We conducted a cross-sectional study on a total of 667 mothers attending primary health centers for vaccines to their children aged two to 18 months. A multistage sampling technique was employed to involve the mothers in the study, and data were collected using a self-administrated open-ended questionnaire. The multivariate linear regression model was used to identify the factors associated with the mothers’ WTP and WTA for vaccines to their children.

**Results:**

The study indicated that 94.2 and 93.1% of the mothers respectively had WTA and WTP values greater than zero, with their corresponding mean values of US$ 6.8 and US$ 4.4. The mothers in the higher monthly household income category, mothers born in the urban areas, and being a female child showed statistically significant positive associations with the mothers’ WTA for the vaccines. While there was a statistically significant positive relationship between monthly household income and the mothers’ WTP; a statistically significant negative relationship exists between the mothers’ age and their WTP for the vaccine to their children.

**Conclusions:**

The findings indicated the mothers’ WTA to WTP ratio of greater than one for the vaccines to their children. The most important factor associated with the mothers’ WTA and WTP was the monthly household income. Thus, improving the socio-economic standards of women in the study area might contribute to reinforcing their immunization services seeking behavior to their children.

## Background

Determining the monetary value of given healthcare services such as vaccinations and their distributions as healthcare resources create challenges to health policy-makers in many health systems, especially to those in the under-resourced countries [1]. Besides, assigning the monetary value of public goods or services is highly complicated because they are devoid of a formal market [2]. However, there existed several methods, including the contingent valuation (CV) method, for eliciting the monetary value of nonmarket goods and services [3–5]. The CV is a direct method of eliciting the monetary value of public goods or services by means of surveys and employing the willingness-to-pay (WTP), which indicates the participants’ maximum amount of money they are willing to give up to obtain the good or service or the willing to accept (WTA), the minimum amount of money that an individual can increase to obtain the attribute [4, 6, 7]. The WTA and WTP measures are also helpful to assess the within-subject disparity on a given attribute [8].

The amount that a person is willing to accept or pay is a rationale decision and is crucial to cost-benefit analysis. However, the WTA is usually considerably higher than the WTP, and this disparity exists even when we use different elicitation methods [6]. The disparity between the WTA and WTP is a well-established measure in behavioral economics or welfare economics [9, 10] and is replicable, universal, and persistently occurring even in participants that have an understanding of the elicitation procedure [11]. Several factors, including the complex psychological processes such as the participants’ attitudes, feelings, familiarity with the target good, risk attitudes, and personality [12] as well as the emotions, and moral perceptions of individuals attached to the attribute [13] contribute to the occurrence of the disparity. The higher value individuals attach to objects they own (WTA) than to objects they do not (WTP) is another source of the disparity [9]. Moreover, people dislike losses more than they value the corresponding gains [14]. The behaviors of individuals, such as loss aversion, that affect the WTP can increase the gap. For substitute goods, the WTA to WTP ratio is smaller, while for health or safety and public or non-market goods, it remains the highest [15]. However, the gap can be turned on and off and can be a source of problematic interpretation [16].

The hypothetical, real, and random real valuation questions can elicit the persons’ WTA and WTP [6]. The values of different attributes, including time, can be generated using either of these approaches [10]. Our study applied the hypothetical valuation method to elicit mothers’ WTA and WTP for vaccines to their children because evidence shows an insignificant difference between the ratios obtained by the real experiments and hypothetical experiments [6]. Besides, the fact that the mothers had fairly equal familiarity with the immunization service and lower misconceptions or misunderstanding about the vaccines contributes to the production of a narrower disparity between the valuations [16, 17].

The government of Iran has introduced the expanded program on immunization (EPI) for children against targeted vaccine-preventable diseases in 1984. Thus, the mothers have the right for their children to freely get the EPI service as it is a public good. Such attributes are best valued using the WTA questions [14]. Accordingly, the child immunization services have been provided by the public health sector free of charge at the health centers of both the urban and rural settings of Iran. Besides, the introduction of the health sector evolution plan in May 2014, resulted in the outsourcing of some of the health centers and provision of the EPI services freely by the private health centers. Currently, the routine EPI targeted ten vaccine-preventable diseases including the provision of the bacillus Calmette-Guerin (BCG) [at birth], oral poliovirus vaccine (OPV) [at birth, 2, 4, 6, 18 months and 6 years of age], hepatitis B (HepB) [at birth, 2 and 6 months], diphtheria, tetanus and pertussis (DTP) [at 6 year of age], pentavalent (DTP + HepB + *Haemophilus influenzae* type b (Hib)) [at 2, 4 and 6 months], and measles-mumps-rubella (MMR) [at 12 and 18 months age] [18]. Additional file 1: Appendix 1 is a summary of the current immunization program in Iran.

The poor economic performance of Iran, especially in recent years due to the sanction plus the limited available public primary health care units to providing EPI services, negatively affected healthcare service provision and use by citizens. Eliciting the WTA and WTP of people for a given good or service in under-resourced countries like Iran can better inform policy-makers not only the monetary value of the good or service but also illuminate the understanding associated with health sector reform such as the immunization service provider shift from public to private. The public goods valuation using the WTA and WTP in the health sector is very rare. Besides, despite our search for different sources, we could not get evidence showing clients’ WTA and WTP for an attribute in Iran. Our study aims to elicit mothers’ WTA and WTP for vaccines to their children in Kermanshah city, western Iran. The findings contribute paramount importance in increasing the knowledge base on WTA and WTA in healthcare in the context and to policy decisions to strengthen the expansion EPI service provision in the private health sectors in Iran and perhaps serve as input in other contexts with similar conditions.

## Methods

### Study setting

We conducted the study in Kermanshah city, a metropolitan and capital city of Kermanshah province, located in western Iran. Iran’s 2016 population census report indicated that the city had a total of 1,083,833 people. Kermanshah city had 50 functional public health centers providing primary healthcare to people, including child immunization services.

### Study design, sample size and sampling method

We conducted a cross-sectional study using the CV method on a total of 700 mothers attending selected health centers of Kermanshah city to elicit their WTA and WTP for vaccines to their 2–18 months old children using a convenience sampling technique. To involve the mothers in the study, we clustered the city into central, western, eastern, southern, and northern geographic areas, and evenly allocated the sample to each area (*n* = 140). Then, after proportionately allocating the sample size of each area to three randomly selected health centers, we collected data from mothers attending the health centers using a self-administered questionnaire during September to December 2019.

### Data collection and variables

The study involved two parts. The first part employed a self-administrated questionnaire to obtain data on socio-demographic characteristics, including the children’s gender, mothers’ age, and educational level, households’ monthly income, and health insurance coverage. The second part of the study elicited the mothers’ open-ended WTA and WTP values for vaccines to their children as recommended in other studies [19, 20]. With the understanding that the EPI service is free of charge, we involved the mothers in the study after getting their *children immunized. We* presented each mother two scenarios and asked open-ended questions to determine her WTA and WTP for the vaccines to her child. *The* WTA *was elicited* by asking the question: *“Had your child not get the vaccines freely today, how much money would you have compensated for the vaccines appropriately?”. Again, we elicited the WTP by posing the question: “Suppose the vaccines were not free of charge and you must pay directly because your child should not miss the vaccines. How much money could you have paid to get the vaccines?”*

In this study, the mothers’ WTA and WTP were the outcome variables, and the children’s sex, mothers’ age in years, birthplace and educational level, and households’ monthly income (in US$) as well as households’ health insurance coverage status were the independent variables. We constructed the questionnaire based on the research objective, and two health economists and an epidemiologist checked for its content validity. Then, we conducted a pilot study on 30 participants in a health center in the study area after amending the opinions of the experts. Once the hypothetical scenarios and the questions were clear and ensured easily understood, we commenced our study.

### Statistical analysis

We used descriptive statistics (percentages, mean with standard deviation (SD), and mean ratios) to characterize the mothers’ WTA and WTP value for the routine EPI vaccines for their children. Besides, because both outcome variables (WTA and WTP) are continuous, we used a multiple linear regression model to assess the association between the dependent and independent variables. The mathematical expression of the model is as follows:

$$ y={\beta}_1+{\beta}_2 age+{\beta}_2{edu}_i+{\beta}_3{hi}_i+{\beta}_5{sex}_i+{\beta}_6{place}_i+{\beta}_7{income}_i+{\mathcal{E}}_i $$

Where ***y*** is the outcome variable (WTA or WTP), ***age*** represents the mother’s age, ***edu*** is educational level of the mother, ***hi*** is the household’s health insurance coverage status, ***sex*** is the child’s sex, ***place*** is the mother’s place of residence, ***income*** is the monthly income of the household, and $\mathbf{\mathcal{E}}$ is the residual.

First, we transformed the mothers’ data with the unwillingness to accept and unwilling to pay values to zero and included in the analysis. Then, we tested the WTP and WTA values for the normality of the distributions of the residuals, and the findings of each model indicated the uneven distribution of the residuals. Then, we further performed a logarithmic transformation of the values and tested for multicollinearity of the variables in the models using the variance inflation factor (VIF). The mean VIF values for the two models (WTP = 1.47 and WTA = 1.45) were less than 10, and we accepted the variables in the models for the analyses [21]. We used the robust for the standard error to minimize the heteroscedasticities.

Second, using the mentioned-above equation and the logarithmic transformed dependent variable, we applied the following log-linear regression model to explore the association between the mothers’ WTP (WTA) and the explanatory variables.

The interpretation of the coefficients of the findings was different from the linear-linear model because the outcome variables were logarithmically transformed, and the explanatory variables were linear. For example, a coefficient of − 0.198 for a variable x indicates that for every one-unit increase in x, the dependent variable increases by about 22% [(exp (0.198) – 1) * 100 = 21.9]. We used the statistical software package Stata version 14.2 for analysis, and the findings were decided statistically significant at the *p*-value of less than 0.05.

## Results

Out of the total sample of 700 mothers, 667 of them had complete data for the analysis, making a response rate of 95.3%. The mothers’ age ranged from 15 to 50 years, with a mean age of 30.8 years and a standard deviation (SD) of 6.6 years. The households with health insurance coverage and mothers with an educational status less than high school accounted for 88.3, and 30%, respectively. More than half of the mothers (52.3%) reported having female children (Table 1).

**Table 1:** Descriptive characteristics of participants included in the study

Sociodemographic variables	n /mean	Percentage/SD
Mother’s mean age (in years)	30.8	6.6
Mother’s education level		
*Less than high school*	200	30.0
*High school and higher*	467	70.0
Health insurance coverage		
*Yes*	589	88.3
*No*	78	11.7
Sex of child		
*Female*	349	52.3
*Male*	318	47.7
Mother’s birthplace		
*Rural*	118	17.7
*Urban*	549	82.3
Monthly household income (US$)		
*< 80*	105	15.7
*80–160*	297	44.5
*160–320*	211	31.6
*> 320*	54	8.10

**Note**: At the time of this study, US$1 was equal to 125,000 Iranian Rilas (IRR)

Overall, 628 (94.2%) mothers had the WTA values of greater than zero, while 39 (5.8%) mothers had the WTA values of zero (holding that the monetary values could not compensate for the vaccines). Furthermore, 621 (93.1%) mothers had the WTP values of greater than zero, and 46(6.9%) mothers had zero values for the reason of “I could not afford the price” in 20(43.5%) and “the vaccines should be free of charge” in the rest 25(56.5%) of mothers]. Generally, 23 (3.4%) mothers had zero value for both the WTA and WTP. The mothers with zero values of the WTP, WTA, and both WTP and WTA accounted for 46(6.9%), 39 (5.8%), and 23 (3.4%), respectively. Overall, 621 (93.1%) mothers had the WTP for the vaccines with values greater than zero, while 46(6.9%) had the WTP values of zero [consisting of 20 (43.5%) mothers with the reasons of “I cannot afford”, which revealed the true zero WTP value, and another 25(56.5%) mothers that hold “the vaccines should be free of charge”, hence, a protested WTP values of zero]. The mean (SD) WTA and WTP values of the mothers for the vaccines accounted for US$ 6.8 (12.6), and US$ 4.3 (9.7), respectively (Table 2). The mean difference between the WTA and WTP values revealed a 36.8% higher WTA value than the WTP value, and the mean WTA to WTP ratio was about 1.6. The mean (SD) WTA and WTP values for mothers in the monthly household income category of less than US$ 80 were 5.2 (10.1) US$ 2.4 (4.6), while their corresponding values for those in the monthly income category of more than US$ 320 were US$ 12.4 (14.1), and US$ 8.1(12.7), respectively.

**Table 2:** The average of WTA and WTP regarding to sociodemographic variables of the samples

Explanatory variables	Mean WTA (SD) in US$ (*n* = 667)	Mean WTP (SD) in US$ (*n* = 667)
Mother’s age (in year)	6.8 (12.6)	4.3 (9.7)
Mother’s educational level		
*Below high school*	6.2 (12.5)	3.4 (8.8)
*High school and above*	7.1 (12.7)	4.7 (10.1)
Health insurance coverage		
*Yes*	6.8 (12.8)	4.4 (9.7)
*No*	6.2 (11.3)	4.0 (10.0)
Sex of the child		
*Female*	7.2 (12.6)	4.7 (10.6)
*Male*	6.3 (12.6)	3.9 (8.6)
Mother’s birthplace		
*Rural*	7.5 (14.2)	3.9 (8.7)
*Urban*	6.6 (12.3)	4.4 (9.9)
Monthly household income (US$)		
*< 80*	5.2 (10.1)	2.4 (4.6)
*80–160*	6.3 (12.7)	4.5 (10.9)
*160–320*	6.8 (13.0)	4.1 (8.6)
*> 320*	12.4 (14.1)	8.1 (12.7)

*WTA* willingness to accept, *WTP* willingness to pay, *SD* standard deviation, *IRR* Iranian Rials. ***Note***: At the time of this study, US$1 was equal to 125,000 IRR

The mothers in the higher monthly household income category, mothers born in the urban areas, and being a female child showed statistically significant positive associations with the logarithm of the mothers’ WTA for the vaccines (Table 3). Holding all other variables constant, the WTA was 300.6% ([3.006 = exp.(1.388)-1]*100) higher for the mothers in the monthly household income category of more than US$ 320 than those in the income category of less than US$ 80. The mothers’ age, and the households’ health insurance coverage did not show any statistically significant association with the mothers’ WTA for the vaccine.

**Table 3:** Ordinary least square regression of the logarithm of WTA of mothers to vaccinate their children (*n* = 667)

Log WTP	Coefficient	Robust S.E	p-value	95% CI
Mothers’ age (in year)	−0.0010	0.0084	0.901	−0.0176 to 0.0155
Mother’s education level				
*Below high school (ref.)*				
*High school and above*	0.083	0.1337	0.533	−0.1792 to 0.3462
Health insurance coverage				
*Yes (ref.)*				
*No*	0.150	0.1736	0.388	−0.1909 to 0.4909
Sex of the child				
*Female (ref.)*				
*Male*	−0.2823^a^	0.1093	0.010	−0.4970 to − 0.0676
Mother’s birthplace				
*Rural (ref.)*				
*Urban*	0.3106^a^	0.1556	0.046	0.0050 to 0.6163
Monthly household income (IRR)				
*< 80*				
*80–160*	0.2201	0.1612	0.173	−0.0965 to 0.5366
*160–320*	0.3602^a^	0.1793	0.045	0.0079 to 0.7125
*> 320*	1.388^a^	0.2509	0.001	0.8955 to 1.8810

**Note**: Dependent variable: logarithm WTA; number of observations: 667; F (8, 658): 5068; Prob>F:0.001; R-squared: 0.0713; S.E: standard error. ^a^Significant at 5% level

Table 4 presents the relationship between the logarithm of the WTP and the explanatory variables included in the regression model. There was a statistically significant negative relationship between the mothers’ age and their WTP for the vaccine to their children. That is, after adjusting for the explanatory variables, the mothers’ WTP for the vaccines decreased by about 1.33% ([− 0.0133 = exp.(− 0.0134)-1]*100) as the mothers’ age increased with each additional year. Besides, there was a statistically significant positive relationship between monthly household income and the mothers’ WTP. The WTP was 200.8% ([2.008 = exp.(1.1013)-1]*100) higher for the mothers in the monthly household income category of more than US$ 320 than those in the income category of less than US$ 80. The WTP did not show any statistically significant association with the mothers’ educational status and birthplace, households’ health insurance coverage, and the children’s sex.

**Table 4:** Multiple linear regression model of the logarithm of WTP of mothers to vaccinate their children (*n* = 667)

Log WTP	Coefficient	Robust S.E	p-value	95% CI
Mother’s age (in year)	−0.0134^a^	0.0071	0.045	−0.0274 to − 0.0002
Mother’s educational level				
*Below high school (ref.)*				
*High school and above*	0.1786	0.1116	0.110	−0.0405 to 0.3978
Health insurance coverage				
*Yes (ref.)*				
*No*	−0.0810	0.1404	0.564	−0.3567 to 0.1947
Sex of the child				
*Female (ref.)*				
*Male*	−0.1113	0.0905	0.219	−0.2890 to 0.0664
Mother’s birthplace				
*Rural (ref.)*				
*Urban*	0.0483	0.1275	0.705	−0.2021 to 0.2987
Monthly household income (in US$)				
< 80				
80–160	0.3338^a^	0.1342	0.013	0.0702 to 0.5974
160–320	0.5145^a^	0.1488	0.001	0.2222 to 0.8068
> 320	1.1013^a^	0.2050	0.001	0.6987 to 1.5040

**Note**: Dependent variable: logarithm WTP; number of observations: 667; F (8, 658): 8.12; Prob>F:0.001; R-squared: 0.0716; S.E: standard error. ^a^significant at 5% level

## Discussion

This study uncovered mothers’ WTA and WTP for vaccines to their children and associated factors. Eliciting the WTA and WTP for healthcare services such as immunization is a vital means of incorporating service users’ preferences in health policy [22]. Besides, the disparity between the WTA and WTP is a useful means to illuminate not only the understanding of values attributed to particular services but also the need for service provision shift from public to private and the users’ possible resistance due to the perceived loss of benefits from the public provider. The proportions of mothers that had the WTA and WTP for the vaccines in our study are considerably higher than the corresponding values reported for a hypothetical vaccine in Malaysia [23]. The WTA to WTP ratio is markedly lower than those reported for health and safety goods, public or non-market goods and even for the ordinary private goods in studies elsewhere. However, whether the elicitation method influenced the observed ratio value or not is not well understood [6].

The use of the WTA and WTP valuation approaches for an already received service can pose a debate. However, these measures are proven capable of providing insights concerning the disparity between the elicited values [2]. The mothers’ mean WTA value of the vaccines in our study is about 1.6 times larger than their WTP. Similarly, others used the same measures of valuation of time, an ever-existing, equally distributed, and immediately consumed private non-market attribute, demonstrated about 1.5–2.0 times larger mean WTA value than the WTP measure [10]. Besides, the mean WTA for nursing consultation value was about 1.5 times larger than the mean WTP value [24].

Studies revealed the mean WTA to WTP ratio values greater than one for different services [2, 4], the mean ratio value (1.55 vs. 3.30) less than the median ratio value, and several factors such as substitute attributes, incomplete information, the cost of goods, and uncertainty attributing to the disparity between these values [2]. The WTA and WTP values and their differences are also subject to the influences of type of vaccines, and disease severity. A vaccine for an acute disease with low mortality and morbidity is likely to have a lower mean WTP value than a chronic disease with high morbidity and mortality [25].

Our findings revealed a higher proportion of WTP and a lower proportion of unwilling mothers to pay for the vaccines to their children (93.1% vs. 3.0%) than the corresponding values (88% vs. 55%) reported from a study in Nigeria [26]. Besides, the mean WTP value in our study is lower than that reported for a hypothetical vaccine in Malaysia [27] and higher than the mean value (US$ 2.08) reported for the Ebola vaccine elsewhere [28]. The WTP of mothers for the Human Papillomavirus vaccine to their daughters (US$ 208) [29] was more than 48 times the mothers’ mean WTP value (US$ 4.3) for the vaccines to their children in our study. The discrepancy between WTA and WTP estimate can increase or decrease based on the perceived risk or benefit of a given attribute [30]. Thus, the possible lower perceived risk associated with the vaccines might have contributed to the lower mean WTP value observed in our study. Besides, the proportion of not WTP mothers (3.0%) in our study is less than one-twelfth of the health professionals reported unwilling to pay for the Hepatitis B virus vaccine in Iran [31], and less than one-eighteenth of those (55%) not WTP for influenza vaccine in China [32]. Generally, the fact that the immunization service is free of any payment might have contributed to the lower proportion of not willing to pay mothers for the vaccines to their children.

The findings of the regression analysis indicated a positive relationship between the mothers’ WTP for vaccinating their children and the monthly income of the households. Both mothers’ mean WTA and WTP values indicated statistically significant associations with the households’ monthly income. The significant association between the mean WTP and household monthly income observed in our study may imply that the open-ended WTP questions are bounded by an individual’s income [14]. Others also stated that the WTA and WTP are explained by income and substitution effects [10]. This finding implies that the wealthier respondents are likely to pay more money to avoid infectious diseases. Others also revealed a positive association of the participants’ WTP for the hepatitis B vaccine with their income level [33]. Furthermore, for self-paid vaccination such as pneumonia and influenza vaccines, the individuals with the highest ability to pay had 18 times higher WTP value than those with the lowest ability to pay [32]. Others reported the monthly income as a key factor affecting the WTP [19]. However, the WTP values considerably vary with income, and the low- and middle-income countries had lower WTP compared to the high-income countries [25]. Besides income, mothers born in the urban areas, and being a female child showed statistically significant positive associations with the mothers’ WTA for the vaccines. There was also a statistically significant negative relationship between mothers’ age and the mothers’ WTP. Others also found that a reduction in the maximum WTP for the self-paid vaccine as age increases [32].

### Limitations of the study

Despite its contribution to the increased knowledge base on the WTA and WTP valuation for an attribute in healthcare and its role for policy action to improve the provision of the EPI services in Iran, our study has some limitations. First, the open-ended questions elicited WTA and WTP monetary values for the vaccines in our study are likely to be under- or overestimated. Second, the cross-sectional analysis does not allow to establish a causal relationship between the explained and explanatory variables. Finally, the study focused on mothers living in one metropolitan city of a province in Iran, and the findings cannot be generalizable to the entire Iran.

## Conclusion

This study elicited evidence concerning mothers’ WTA and WTP for vaccines to their 2 to 18 months old children in Kermanshah city, western Iran. The findings indicated a high proportion of mothers WTP for the vaccines to their children, and there existed statistically significant associations between the mothers’ WTP, and households’ monthly income, and the mothers’ age. The mothers’ mean WTA was also significantly associated with the households’ income, children’s sex, and mothers’ birthplace. The mothers’ WTA and WTP for the vaccines to their children observed in our findings contribute paramount importance input for policy-makers to reinforce primary health care services including the expansion of EPI services in the private sector.

## Supplementary information

**Additional file 1.** Appendix 1: Current national childhood immunization program of Iran (May 2020).

**Additional file 2.** Participant Information Sheet and Informed Consent Form.

## Data Availability

The data used for the analysis in this study are available from the corresponding author upon reasonable request.

## Abbreviations

CV: Contingent valuation
EPI: Expanded program on immunization
WTA: Willingness to accept
WTP: Willingness to pay
