# Supplementary material for: Mothers’ willingness to accept and pay for vaccines to their children in western Iran: a contingent valuation study
Source: BMC Pediatr. 2020 Jun 23;20:307. doi: 10.1186/s12887-020-02208-4 (PMC7310440; doi:10.1186/s12887-020-02208-4)
Supplement: Supplementary file 1 — Additional file 1. Appendix 1: Current national childhood immunization program of Iran (May 2020). [file 12887_2020_2208_MOESM1_ESM.docx]

| Type of vaccine | Schedule |
| --- | --- |
| BCG | Birth |
| OPV | Birth, 2, 4, 6, 18 months and 6 years |
| HepB | Birth, 2, and 6 months |
| MMR | 12 and 18 months |
| Pentavalent | 2, 4 and 6 months |
| DTP | 6 years |

**Appendix 1**: Current national childhood immunization program of Iran (May 2020)
