# Supplementary material for: Mothers’ willingness to accept and pay for vaccines to their children in western Iran: a contingent valuation study
Source: BMC Pediatr. 2020 Jun 23;20:307. doi: 10.1186/s12887-020-02208-4 (PMC7310440; doi:10.1186/s12887-020-02208-4)
Supplement: Supplementary file 2 — Additional file 2. Participant Information Sheet and Informed Consent Form. [file 12887_2020_2208_MOESM2_ESM.docx]

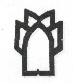
**Kermanshah University of Medical Sciences**

**Participant Information Sheet and Informed Consent Form**

My name is __________________. I am an academic staff of the Kermanshah University of Medical Sciences (KUMS). I kindly request you to lend me your attention to explain you about the study and to participate in the study.

**The research title:** *Mothers’ willingness to accept pay and pay accept for vaccines to their 2 -18 months old children in western Iran: A contingent valuation study*

**Aim of the study:**

This study aims to assess the willingness to accept (WTA) and pay (WTP) of mothers attending the primary health centers for vaccines to their children during 2019 in Kermanshah city, western Iran.

**Procedure and duration:**

I want to inform you that the study involves completing self-administered questionnaire to assess the sociodemographic characteristics and an interview to elicit the WTA and WTP value for vaccines. Both steps will take about 15 to 20 minutes. Hence, I kindly request you to spare me this time for the study.

**Risks and benefits:**

This study is non-experimental and the risk of being participating in the study is minimal. However, it will take few minutes from your time. There will not be any direct payment, promotion or reward and even food to be served for participating in this study while the findings of the study are expected to be of paramount importance for the decision-makers to improve the primary health care including the provision of the immunization services in Iran.

**Confidentiality:**

The information you provide will be confidential. Even there will no need of mentioning your name and related identifiers. The findings of the study will be generally focusing on purposively selected public health centers in Kermanshah city. No reference will be made in oral or written reports that can link participants to the information obtained.

**Rights:**

The participation for the interview is fully voluntary. You have the right to declare to participate or not in this study. If you decide to participate, you have the right to withdraw from completing the questionnaire as well as from the interview process at any point you like without any explanation and this will not label you for any loss of the benefits that you otherwise are entitled from the service. If you decide to participate in the study, you have the right to ask questions for clarifications and get satisfactory answer. Besides, you do not have to answer any question that you do not want to answer.

**Contact address:**

If there are any questions or enquires any time about the study, please contact:

- Dr Satar Rezaei : +98 …….

For any ethical related issues or concerns in the conduct of the study, please contact the ethics committee of the Deputy of Research, Kermanshah University of Medical Sciences chairperson:

- Dr. _____________ : +98……….

**Declaration of informed voluntary consent:**

I have read/it was read to me the participant information sheet. I have clearly understood the purpose of the study, the procedures, the risks and benefits, issues of confidentiality, the rights of participating and the contact address for any questions. I have been given the opportunity to ask questions for things that may have been unclear and get satisfactory answer. I was informed that I have the right to withdraw from the study at any time or not to answer any question that I do not want. Therefore, I declare my voluntary consent to participate in this study with my signature as indicated below. _________________________

**SECTION I**

**Socioeconomic characteristics of the participant: Please write your response or put tick mark on the space provided as appropriate.**

1. Mother’s age (in years) ……………….
2. Mother’s birthplace

Urban Rural

1. Sex of the child

Male Female

1. Educational status

Below high school High school and above

1. Health insurance coverage

Yes No

1. Monthly household income in Iranian Rials (IRR)

Less than 10000000 IRR

Between 10000000 and 20000000 IRR

20000000 to 40000000 IRR

More than 40000000 IRR

**SECTION II**

This section included two scenarios: Eliciting the willingness to accept (WTA) and willingness to pay (WTP) measures for vaccines to children.

**Scenario 1.** Eliciting the willingness to pay (WTA) measures: Imagine that there is no vaccination service in the public health care system. Your child should get the vaccines today, and you have to pay directly to get the vaccine.” “Had your child not get the vaccines freely today, how much money would you have compensated for the vaccines appropriately?”

1. Would you accept to pay for the vaccination to your child ? Yes No
   1. If your response is “NO”, please state the main reason for not willing to pay for the vaccines: ………………………………………..
   2. If your response is YES, and had your child not get the vaccines freely today, how much money would you have compensated for the vaccines appropriately? ……………………

**SECTION II**

**Scenario 2.** Eliciting the willingness to pay (WTP) measures :

“Suppose the vaccines were not free of charge and you must pay directly because your child should not miss the vaccines.”

Would you be willing to pay for the vaccination? Yes No

If your response is NO; please stated the mean reason below: …………………………………….

If your response is YES, how much money *(in Iranian Rials)* could you have paid to get the vaccines? ………………………………

Thank you for taking the time.
